# Exploring the Molecular Mechanism of Qing Guang An Granule in Treating Glaucoma Using Network Pharmacology and Molecular Docking

**DOI:** 10.1155/2020/8824150

**Published:** 2020-11-23

**Authors:** Chen Ou, Houpan Song, Yasha Zhou, Jun Peng, Qinghua Peng

**Affiliations:** Hunan University of Chinese Medicine, Changsha, Hunan 410208, China

## Abstract

**Background:**

Qing Guang An Granule (QGAG), a Chinese patent medicine, has been used clinically to treat glaucoma for more than 20 years.

**Objective:**

To explore the possible mechanism of treatment of QGAG in glaucoma by using network pharmacology and molecular docking in this study.

**Methods:**

Active compounds and targets of each herb in QGAG were retrieved via the Traditional Chinese Medicine Systems Pharmacology Database and Analysis Platform (TCMSP). Glaucoma-related targets were acquired from OMIM and DisGeNET database. Key targets of QGAG against glaucoma were acquired by overlapping the above targets via the Venn diagram. Using the DAVID, Gene Ontology (GO) enrichment analysis and Kyoto Encyclopedia of Genes and Genomes (KEGG) pathway analysis of the key targets were performed. The docking process was performed using the AutoDock 4.2.6 and AutoDock Vina 1.1.2.

**Results:**

The 55 active compounds and 173 targets were obtained and constructed a compound-target network. The 20 key targets of QGAG in treating glaucoma were acquired, and these targets are involved in the apoptotic process, cellular response to hypoxia, negative regulation of cell growth, and ovarian follicle development. The main pathways are p53, HIF-1, PI3K-Akt, and neurotrophin signaling pathway.

**Conclusion:**

QGAG may exert a protective effect by acting on the optic nerve at a molecular and systemic level. This study can provide a certain basis for future researches on exploring the QGAG in treating glaucoma and provide new ideas for developing new drugs.

## 1. Introduction

Glaucoma is the second leading cause of blindness in the world characterized by optic atrophy and visual field defects [1]. It is estimated that there are more than 10 million patients with blindness worldwide due to glaucoma [2]. The primary treatment for glaucoma is to reduce intraocular pressure by pressure lowering eye drops, laser treatment, and surgery [3]. However, the pathophysiology of glaucoma is considered to be multifactorial and complex, single targeted therapies may not be sufficient enough and may induce inevitable side effects and toxicity. Therefore, the discovery of new multitarget drugs is necessary.

Traditional Chinese medicine (TCM) has been used to treat glaucoma for thousands of years. Shennong's Classic of Materia Medica (Shennong Bencao Jing), one of the four classics of TCM, has first recorded glaucoma. In TCM, glaucoma is considered as a “five wind cataract”, which is caused by emotional depression, qi movement stagnation, and liver fire flaming upward [4]. In recent years, the prevention and treatment of glaucoma using TCM has attracted increasing attention [5]. Oral administration is one of the main ways of Chinese medicine application, with unique advantages and broad prospects for development.

Qing Guang An Granule (QGAG), a Chinese patent medicine, consists of eight medicine herbs, including *Astragalus membranaceus* (Huangqi, HQ), *Plantago* (Cheqianzi, CQZ), Safflower (Honghua, HH), *Poria cocos* (Fuling, FL), Radix Paeoniae Rubra (Chishao, CS), *Atractylodes macrocephala* (Baizhu, BZ), *Rehmannia glutinosa* (Shengdihuang, SDH), and Earthworm (Dilong, DL). QGAG, first proposed by Professor Qinghua Peng, has been used clinically to treat glaucoma for more than 20 years. Huangqi (HQ) and Shengdihuang (SDH) benefit qi and nourish yin, Dilong (DL), Honghua (HH) and Chishao (CS) activate blood circulation, and Fuling (FL), Baizhu (BZ), and Cheqianzi (CQZ) remove dampness [6]. Previous experiments have indicated that QGAG can reduce intraocular pressure of glaucoma [7]. The mechanism of QGAG against glaucoma is related to the downregulated expression of TGF-*β*1 and Smad3 and reduced scar tissue hyperplasia [8]. However, each Chinese medicine contains complex chemical components, and its efficacy is not simply added up. Therefore, further study of the molecular mechanisms of QGAG in treating glaucoma has great clinical significance.

Network pharmacology is an approach for exploring the potential pharmacological mechanism of TCM through constructing biological networks. TCM has the features of multi-ingredients and multitargets, while network pharmacology has the features of multipathways and multilevels. Therefore, the systematic evaluation method of network pharmacology has a good predictive effect in the study of TCM [9]. Molecular docking is a computational method that predicts the binding interaction of the small molecule ligands and the target proteins, and it can be applied to confirm and check the result of network pharmacology [10]. In this study, we aim to explore the relationships between QGAG and glaucoma through network pharmacology and molecular docking.

## 2. Methods

### 2.1. Collection of Active Compounds and Targets in QGAG

Active compounds of each herb in QGAG were retrieved via the Traditional Chinese Medicine Systems Pharmacology Database and Analysis Platform (TCMSP, http://tcmspw.com/tcmsp.php); duplicate compounds were removed. Oral bioavailability (OB) ≥ 30% and drug-likeness (DL) index ≥ 0.18 are considered as important pharmacokinetic parameters to filter active compounds [11]. Prediction of possible targets related to compounds of QGAG was performed by searching “Related Targets” of the TCMSP Database. The obtained targets were entered into STRING (https://string-db.org/) to transform gene symbols.

### 2.2. Collection of Known Glaucoma-Related Targets

Glaucoma-related targets were acquired from the OMIM database (https://omim.org) and DisGeNET database (http://www.disgenet.org/). The obtained targets were entered into STRING (https://string-db.org/) to build Protein-Protein Interaction (PPI) networks.

### 2.3. Key Targets of QGAG for Treating Glaucoma

The key targets of QGAG against glaucoma were acquired by overlapping the above targets via Venn diagram (http://bioinfogp.cnb.csic.es/ tools/venny/index.html).

### 2.4. Network Construction

Based on the key targets obtained from the Venn diagram, we constructed an interaction network of the herbs, compounds, disease, and key targets using Cytoscape 3.6.0.

### 2.5. Gene Ontology (GO) Enrichment Analysis and Kyoto Encyclopedia of Genes and Genomes (KEGG) Pathway Analysis

GO enrichment analysis is normally classified into cell component (CC), biological process (BP), and molecular functions (MF) categories [12]. Using the DAVID (https://david.ncifcrf.gov/), the GO enrichment analysis and KEGG pathway analysis of the key targets were performed to obtain the related functions and pathways.

### 2.6. Molecular Docking

The three-dimensional structures of active compounds were retrieved from PubChem (https://pubchem.ncbi.nlm.nih.gov/), and the receptor proteins of key targets were obtained from Protein Data Bank (http://www.rcsb.org/pdb). The receptor proteins removed ligands and water by using the protein visualization software PyMOL 2.3.4. The docking process was performed using the AutoDock 4.2.6 and AutoDock Vina 1.1.2. From the docking results, docked conformation with the best scoring was selected for further mapping analysis. PyMOL was performed to show the interactions between receptor protein and ligand.

## 3. Results

### 3.1. The Active Compounds and Targets in QGAG

**Figure 1:**
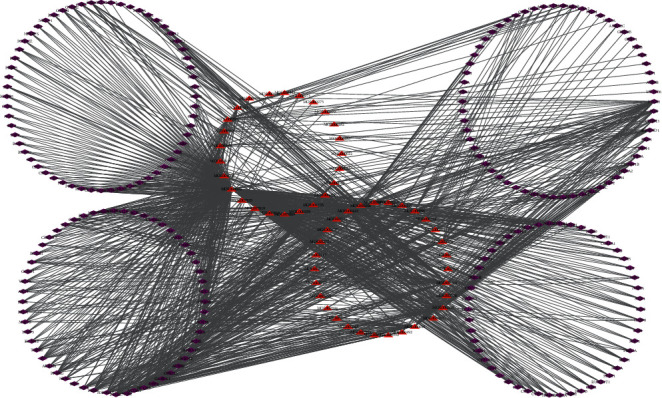
The compound-target network (red triangle represents active compounds; purple diamond represents targets).

QGAG consists of HQ, CQZ, HH, FL, CS, BZ, SDH, and DL. The 17 components of HQ, 7 components of CQZ, 17 components of HH, 6 components of FL, 14 components of CS, 4 components of BZ, 3 components of SDH, and 2 components of DL were obtained from the TCMSP. All active components should satisfy the criteria of OB ≥ 30% and DL ≥ 0.18. The 55 active compounds were obtained after deleting duplicates. The obtained compounds from the eight medicine herbs are shown in Table 1. The obtained compounds were linked to targets, and networks of compound-target interactions were plotted using Cytoscape 3.6.0 software. The resulting compound-target network is shown in Figure 1. The network contains 283 nodes and 1194 compound-target interactions. For all active compounds, quercetin, kaempferol, luteolin, 7-O-methylisomucronulatol, baicalein, beta-sitosterol, Stigmasterol, formononetin, and isorhamnetin have the highest degree distributions.

### 3.2. PPI Network of Glaucoma-Related Targets

**Figure 2:**
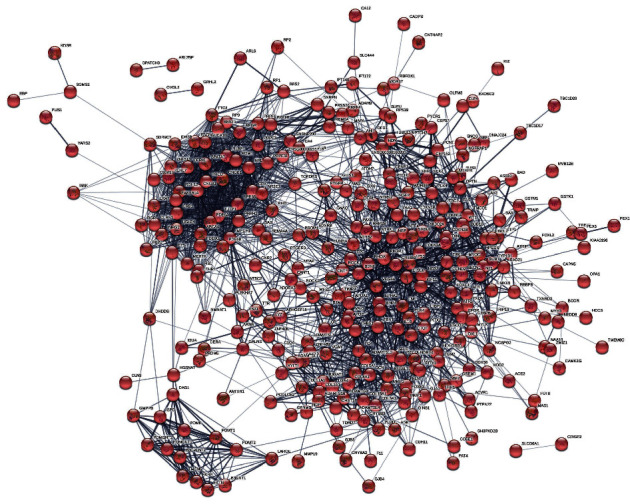
The PPI network of glaucoma-related targets.

By researching the OMIM and DisGeNET database, 176 and 380 genes were acquired in these two databases, respectively. A total of 345 nodes and 2630 edges were involved in the PPI network, with an average of 15.2 edges per node. The resulting PPI network is shown in Figure 2.

### 3.3. Key Targets of QGAG for Treating Glaucoma

**Figure 3:**
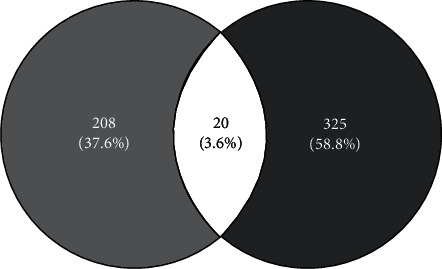
Overlapping the 228 targets of active compounds and 345 targets of related glaucoma.

The key targets of QGAG against glaucoma were obtained by overlapping the 228 targets of active compounds and 345 targets of related glaucoma with a Venn diagram. This analysis derived 20 targets, including ADRB2, RHO, MMP3, VEGFA, BCL2, CDKN1A, BAX, TP53, MMP1, GJA1, IL1B, SELE, PTGER3, NOS3, CYP1B1, GSTP1, COL3A1, GSTM1, TDRD7, and LYZ. The resulting Venn diagram is shown in Figure 3.

### 3.4. Network between Medicine Herbs, Active Compounds, Key Targets, and Glaucoma

**Figure 4:**
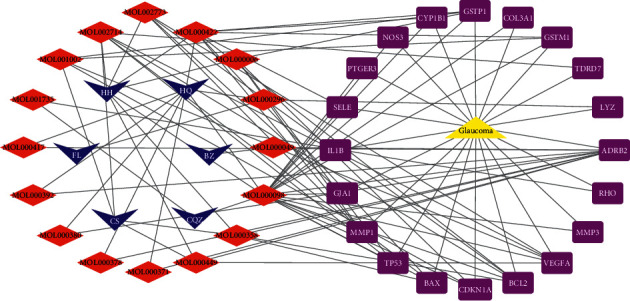
The herbs-active compounds-key targets-disease network (blue ellipse represents medicine herbs; red diamond represents active compounds; purple round rectangle represents key targets; yellow triangle represents disease).

The resulting medicine herbs-active compounds-key targets-disease network is shown in Figure 4. Thus, 20 key targets related to 16 active compounds of QGAG were selected against glaucoma. Among them, the key target with the most connections with active compounds is ADRB2.

### 3.5. GO Enrichment Analysis and KEGG Pathway Analysis

To further explore the multiple mechanisms of QGAG on glaucoma from a systematic level, GO enrichment analysis and KEGG pathway enrichment of the 20 key targets were performed. Among them, 9 items related to biological processes were found, including response to a toxic substance, positive regulation of intrinsic apoptotic signaling pathway, apoptotic process, cellular response to hypoxia, and ovarian follicle development. Meanwhile, 5 items were related to cell composition, including mitochondrion, mitochondrial outer membrane, endoplasmic reticulum, endoplasmic reticulum membrane, and membrane, and 4 items were related to molecular function, including identical protein binding, protein heterodimerization activity, ubiquitin-protein ligase binding, and protein homodimerization activity. The GO terms of each category are illustrated in Table 2.

**Figure 5:**
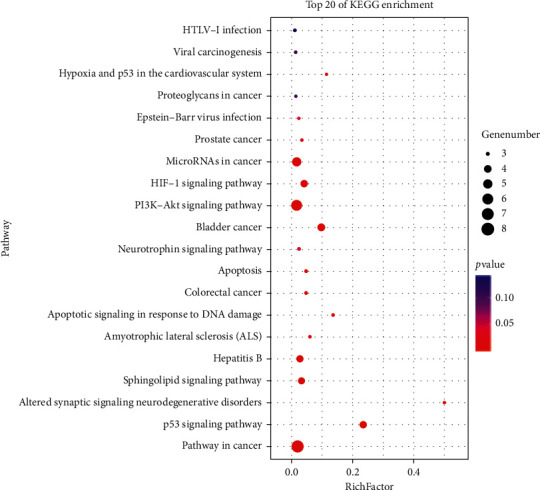
Bubble diagram of KEGG pathway analysis. The *Y*-axis label represents the pathway and the *X*-axis label represents the rich factor. The size and color of the bubble represent the number of genes enriched in pathway and enrichment significance, respectively.

The pathways of 20 key targets were analyzed via pathway enrichment, and 20 signaling pathways were obtained. The involved pathway was mainly composed of the p53 signaling pathway, PI3K-Akt signaling pathway, HIF-1 signaling pathway, Sphingolipid signaling pathway, apoptosis, and neurotrophin signaling pathway (Figure 5).

### 3.6. Molecular Docking

**Figure 6:**
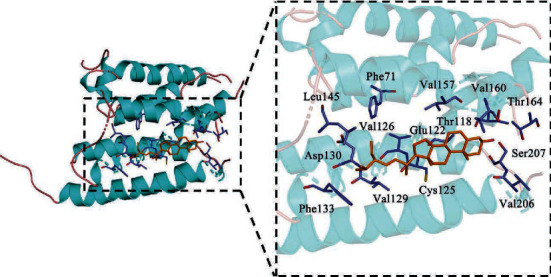
Molecular docking between Stigmasterol and ADRB2.

The molecular docking between active compounds quercetin, kaempferol, luteolin, 7-O-methylisomucronulatol, baicalein, beta-sitosterol, Stigmasterol, formononetin, isorhamnetin, and key target ADRB2 (PDB ID: 2R4S) was performed. The binding energies of these 9 active compounds with ADRB2 are −7.8, −7.9, −8.0, −7.6, −7.9, −9.2, −9.5, −7.5, and −7.9 kcal/mol, respectively. The more negative the binding energy, the more stable the compound binds to the target. The Stigmasterol binding with the ADRB2 showed the highest binding energy (−9.5 kcal/mol).  Figure 6 shows the binding between Stigmasterol and ADRB2. The amino acid residues Leu145, Phe71, Val157, VAL160, Thr164, Ser207, Val206, Cys125, Val129, Phe133, Asp130, Val126, Glu122, and Thr118 participated in the hydrophobic interactions between Stigmasterol small molecule ligand.

## 4. Discussion

The efficacy of QGAG has been verified through more than 20 years of clinical application [13]. However, the pharmacological mechanism of QGAG in treating glaucoma still lacks deep understanding. In this study, we used the network pharmacology and molecular docking approach to illuminate the multiple underlying mechanisms of QGAG in glaucoma treatment from a systematic perspective. The active compounds, targets, PPI networks, GO enrichment analysis, and KEGG pathway analysis were used to reveal the relationships between QGAG and glaucoma.

TCMSP database was built based on the framework of systems pharmacology for herbal medicines. It consists of all the 499 herbal medicines registered in the Chinese pharmacopeia with 29,384 ingredients, 3,311 targets, and 837 related diseases [11]. The 55 active compounds and 228 targets were obtained via TCMSP databases and constructed a compound-target network. The key compounds of QGAG in the compound-target network included quercetin, kaempferol, luteolin, 7-O-methylisomucronulatol, baicalein, beta-sitosterol, Stigmasterol, formononetin, and isorhamnetin. Stigmasterol is a phytosterol and possesses anti-inflammatory, antioxidant, and anticancer activities [14]. Glaucomatous blindness is caused by the eventual apoptosis of retinal ganglion cells, and quercetin has been proven to protect retinal ganglion cells [15]. Some studies have shown that quercetin can inhibit the proliferation of human fibroblasts and thus reduce postoperative scarring of the filtration tract [16, 17]. In a recent study, it has been reported that baicalein can promote the growth of retinal ganglion axons and protect optic nerve activity, improving the microcirculation of the visual nipple [18].

In our study, there are 20 key targets of QGAG in treating glaucoma. RHO has a close relationship with glaucoma, and the inhibition of RHO expression can lower intraocular pressure, change retinal vasculature perfusion, and promote regeneration of the optic nerve [19]. BCL and BAX are members of the BCL-2 family, which relieve optic nerve injury by preventing apoptosis of retinal ganglion cells [20]. Presently, TDRD7, CYP1B1, GJA1, IL1B, MMP1, and MMP3 have been considered as a causative gene for glaucoma [21–25]. ADRB2, a gene of *β*-adrenergic receptor family genes, is widely distributed in eye tissue [26]. Timolol maleate is a *β*-adrenergic receptor antagonist that has been widely used as eye drops for the treatment of high intraocular pressure in patients with glaucoma for many years. The result of molecular docking has shown that the binding energies of selected active compounds with ADRB2 lower than −7 kcal/mol indicate that these active compounds might act directly on ADRB2 to treat glaucoma.

GO enrichment analysis and KEGG pathway mapping are efficient methods to analyze gene function. GO enrichment analysis found that the effects of QGAG in the treatment of glaucoma are mainly reflected in the apoptotic process, cellular response to hypoxia, negative regulation of cell growth, and ovarian follicle development. Enriched KEGG pathways were predominantly associated with some signaling pathways. The PI3K-Akt and P53 signaling pathway are related to apoptosis, and downregulating the expression of P53 can reduce ganglion cell apoptosis [27]. A study has indicated that HIF-1 expression in hypoxic conditions in glaucoma might be a crucial stage in damage to optic nerve axon and retinal ganglion cells [28]. Inflammation might be involved in the pathogenesis of glaucoma, and the sphingolipids signaling pathway plays an important signaling role in the pathophysiology of ocular inflammatory diseases [29]. Neurotrophins as a therapeutic drug for glaucoma and neurotrophin signaling pathway have been verified as vital signaling to protect the optic nerve [30]. Based on the results of this study, the abovementioned active compounds, targets, and pathways are associated with the pharmacological mechanisms of QGAG in glaucoma treatment.

## 5. Conclusion

In summary, we used the network pharmacology and molecular docking approach to illuminate the multiple underlying mechanisms of QGAG in glaucoma treatment. The results indicate that QGAG may exert a protective effect by acting on retinal ganglion cell and optic nerve at a molecular level. However, the results require further experimental verification. This study can provide a certain basis for future research on exploring the QGAG in treating glaucoma and provide new ideas for developing new drugs.

## Figures and Tables

**Table 1:** The active compounds from the eight medicine herbs.

Code	Molecule name	OB%	DL	Herb name
M1	Mairin	55.38	0.78	HQ
M2	Jaranol	50.83	0.29	HQ
M3	Hederagenin	36.91	0.75	HQ/FL
M4	(3S, 8S, 9S, 10R, 13R, 14S, 17R)-10,13-dimethyl-17-[(2R, 5S)-5-propan-2-yloctan-2-yl]-2, 3, 4, 7, 8, 9, 11, 12, 14, 15, 16, 17-dodecahydro-1H-cyclopenta[a]phenanthren-3-ol	36.23	0.78	HQ/BZ
M5	Isorhamnetin	49.60	0.31	HQ
M6	3,9-di-O-methylnissolin	53.74	0.48	HQ
M7	7-O-methylisomucronulatol	74.69	0.30	HQ
M8	9,10-Dimethoxypterocarpan-3-O-*β*-D-glucoside	36.74	0.92	HQ
M9	(6aR,11aR)-9,10-dimethoxy-6a,11a-dihydro-6H-benzofurano [3,2-c]chromen-3-ol	64.26	0.42	HQ/DL
M10	Bifendate	31.10	0.67	HQ
M11	Formononetin	69.67	0.21	HQ
M12	Calycosin	47.75	0.24	HQ
M13	Kaempferol	41.88	0.24	HQ/HH/SDH
M14	FA	68.96	0.71	HQ
M15	Isomucronulatol-7, 2′-di-O-glucosiole	49.28	0.62	HQ
M16	1, 7-Dihydroxy-3, 9-dimethoxy pterocarpene	39.05	0.48	HQ
M17	Quercetin	46.43	0.28	HQ/CQZ/HH/SDH
M18	Dinatin	30.97	0.27	CQZ
M19	Sitosterol	36.91	0.75	CQZ/CS
M20	Daucostero_qt	36.91	0.75	CQZ
M21	Dihydrotricetin	58.12	0.28	CQZ
M22	Hypolaetin	33.24	0.28	CQZ
M23	orobanchoside_qt	55.99	0.82	CQZ
M24	Poriferast-5-en-3beta-ol	36.91	0.75	HH
M25	4-[(E)-4-(3, 5-dimethoxy-4-oxo-1-cyclohexa-2, 5-dienylidene)but-2-enylidene]-2, 6-dimethoxycyclohexa-2, 5-dien-1-one	48.47	0.36	HH
M26	Lignan	43.32	0.65	HH
M27	Pyrethrin II	48.36	0.35	HH
M28	6-Hydroxykaempferol	62.13	0.27	HH
M29	Baicalein	33.52	0.21	HH/CS
M30	qt_carthamone	51.03	0.20	HH
M31	Quercetagetin	45.01	0.31	HH
M32	7, 8-Dimethyl-1H-pyrimido[5,6-g]quinoxaline-2, 4-dione	45.75	0.19	HH
M33	Beta-carotene	37.18	0.58	HH
M34	Baicalin	40.12	0.75	HH/CS
M35	Beta-sitosterol	36.91	0.75	HH/CS
M36	Stigmasterol	43.83	0.76	HH/CS
M37	Luteolin	36.16	0.25	HH/SDH
M38	CLR	37.87	0.68	HH
M39	(2R)-2-[(3S, 5R, 10S, 13R, 14R, 16R, 17R)-3,16-dihydroxy-4, 4, 10, 13, 14-pentamethyl-2, 3, 5, 6, 12, 15, 16, 17-octahydro-1H-cyclopenta[a]phenanthren-17-yl]-6-methylhept-5-enoic acid	30.93	0.81	FL
M40	Trametenolic acid	38.71	0.80	FL
M41	Cerevisterol	37.96	0.77	FL
M42	Ergosta-7, 22E-dien-3beta-ol	43.51	0.72	FL
M43	Ergosterol peroxide	40.36	0.81	FL
M44	Ellagic acid	43.06	0.43	CS
M45	Paeoniflorgenone	87.59	0.37	CS
M46	Paeoniflorin	53.87	0.79	CS
M47	Spinasterol	42.98	0.76	CS
M48	(+)-catechin	54.83	0.24	CS
M49	(2R,3R)-4-methoxyl-distylin	59.98	0.30	CS
M50	Stigmast-7-en-3-ol	37.42	0.75	CS/DL
M51	Ethyl oleate (NF)	32.40	0.19	CS
M52	Campest-5-en-3beta-ol	37.58	0.71	CS
M53	14-Acetyl-12-senecioyl-2E,8Z,10E-atractylentriol	63.37	0.30	BZ
M54	3*β*-acetoxyatractylone	54.07	0.22	BZ
M55	8*β*-ethoxy atractylenolide III	35.95	0.21	BZ

**Table 2:** The GO enrichment analysis.

Category	Description	Cout	Pop hits	*P* value	Fold enrichment
BP	Response to toxic substance	5	85	2.2*E* − 6	49.39
	Response to gamma radiation	3	31	5.5*E* − 4	81.25
	Positive regulation of intrinsic apoptotic signaling pathway	3	33	6.3*E* − 4	76.33
	Negative regulation of the apoptotic process	5	455	1.5*E* − 3	9.23
	Apoptotic process	5	567	3.3*E* − 3	7.40
	Cellular response to hypoxia	3	96	5.2*E* − 3	26.24
	Negative regulation of cell growth	4	121	3.3*E* − 4	27.76
	Cellular response to DNA damage stimulus	3	208	2.3*E* − 2	12.11
	Ovarian follicle development	3	42	1.0*E* − 3	59.97

CC	Mitochondrion	6	1331	1.0*E* − 2	4.11
	Mitochondrial outer membrane	3	149	1.0*E* − 2	18.35
	Endoplasmic reticulum membrane	4	862	5.8*E* − 2	4.23
	Endoplasmic reticulum	3	828	2.1*E* − 1	3.30
	Membrane	3	2200	6.9*E* − 1	1.24

MF	Identical protein binding	5	749	8.7*E* − 3	5.63
	Protein heterodimerization activity	4	465	1.4*E* − 2	7.26
	Ubiquitin protein ligase binding	3	287	4.1*E* − 2	8.82
	Protein homodimerization activity	5	730	8.0*E* − 3	5.78

## Data Availability

We have presented all our main data in the form of figures and an additional file. The data will be available upon request.

## References

[1] Jonas J. B., Aung T., Bourne R. R., Bron A. M., Ritch R., Panda-Jonas S. (2017). Glaucoma. *The Lancet*.

[2] Keel S., Xie J., Foreman J. (2019). Prevalence of glaucoma in the Australian national eye health survey. *British Journal of Ophthalmology*.

[3] Bucolo C., Platania C. B. M., Drago F. (2018). Novel Therapeutics in glaucoma management. *Current Neuropharmacology*.

[4] Peng Q. H. (2016). *Ophthalmology of Traditional Chinese Medicine*.

[5] Liu H. J., Li X., Zhang Z. D. (2020). Efficacy and safety of Bujing Yishi tablet for glaucoma with controlled IOP: study protocol for a multi-centre randomized controlled trial. *Trials*.

[6] Wang M., Shen B. B., Luo J. (2015). Progress in the studies of compound qingguangan. *Asia-Pacific Traditional Medicine*.

[7] Xiang Y., Liu J. Q., Li Q. (2018). Effect of qingguangan granules on intraocular pressure of spontaneous glaucoma mice. *Journal of Hunan University of Chinese Medicine*.

[8] Tan H. Y., Peng Q. H., Li W. J. (2016). Effects of active ingredients of Qingguan’an granules on the expression of TGF-*β*1 and Smad3 in scarring tissues of filtering tract after filtering surgery in rabbits. *China Journal of Traditional Chinese Medicine and Pharmacy*.

[9] Song Y. J., Bao J. M., Zhou L. Y. (2020). An analysis of the anti-neuropathic effects of qi she pill based on network pharmacology. *Evidence Based Complementary and Alternative Medicine*.

[10] Tao Y. G., Huang X. F., Wang J. Y. (2020). Exploring molecular mechanism of huangqi in treating heart failure using network pharmacology. *Evidence Based Complementary and Alternative Medicine*.

[11] Ru J. L., Li P., Wang J. N. (2014). TCMSP: a database of systems pharmacology for drug discovery from herbal medicines. *J Cheminform*.

[12] Jain A., Kihara D. (2019). NNTox: gene ontology-based protein toxicity prediction using neural network. *Science Reports*.

[13] Peng Q. H., Luo P., Li C. K. (1997). Clinical study on the effect of Qingguangan Granule on postoperative glaucoma patients. *China Journal of Chinese Ophthalmology*.

[14] Wang S., Sun Y., Li C. M. (2019). Research progress of stigmasterol. *China Pharmaceuticals*.

[15] Cheng Y., Wang L., Tian Y. (2019). Protective effects of quercetin on N-methyl-D-aspartic acid induced retinal ganglion cells injury and its mechanisms. *Recent Advances in Ophthalmology*.

[16] Cao H., He X. T., Xiang Y. Q. (2016). Effect of quercetin on the proliferation of scar fibroblasts cultured in vitro after glaucoma surgery. *Shanxi Medical Journal*.

[17] Wang T., Li B. Q., Wang J. J. (2007). Inhibitory effect of quercetin on human Tenon’s capsule fibroblasts. *Recent Advances in Ophthalmology*.

[18] Ruan J. Q., Li Q. Y., Hu M. L. (2019). Advances in research on the treatment of glaucoma with baicalin. *Journal of Jilin Medical College*.

[19] Schehlein E. M., Robin A. L. (2019). Rho-associated kinase inhibitors: evolving strategies in glaucoma treatment. *Drugs*.

[20] Phatak N. R., Stankowska D. L., Krishnamoorthy R. R. (2016). Bcl-2, Bcl-xL, and p-AKT are involved in neuroprotective effects of transcription factor Brn3b in an ocular hypertension rat model of glaucoma. *Molecular Vision*.

[21] Lachke S. A., Alkuraya F. S., Kneeland S. C. (2011). Mutations in the RNA granule component TDRD7 cause cataract and glaucoma. *Science*.

[22] Alsubait A., Aldossary W., Rashid M., Algamdi A., Alrfaei B. M. (2020). CYP1B1 gene: implications in glaucoma and cancer. *Journal of Cancer*.

[23] Huang X., Wang N., Xiao X., Li S., Zhang Q. (2015). A novel truncation mutation in GJA1 associated with open angle glaucoma and microcornea in a large Chinese family. *Eye*.

[24] Tsironi E. E., Pefkianaki M., Tsezou A. (2009). Evaluation of MMP1 and MMP3 gene polymorphisms in exfoliation syndrome and exfoliation glaucoma. *Molecular Vision*.

[25] Mookherjee S., Banerjee D., Chakraborty S. (2010). Association of IL1A and IL1B loci with primary open angle glaucoma. *BMC Med, Genet*.

[26] Litonjua A. A., Gong L., Duan Q. L. (2010). Very important pharmacogene summary ADRB2. *Pharmacogenetics and Genomics*.

[27] Abraham A. G., O’Neill E. (2014). PI3K/Akt-mediated regulation of p53 in cancer. *Biochemical Society Transactions*.

[28] Reszeć J., Zalewska R., Bernaczyk P., Chyczewski L. (2012). HIF-1 expression in retinal ganglion cells and optic nerve axons in glaucoma. *Folia histochemica et cytobiologica*.

[29] Grambergs R., Mondal K., Mandal N. (2019). Inflammatory ocular diseases and sphingolipid signaling. *Bioactive Ceramides in Health and Disease*.

[30] Chitranshi N., Dheer Y., Abbasi M., You Y., Graham S. L., Gupta V. (2018). Glaucoma pathogenesis and neurotrophins: focus on the molecular and genetic basis for therapeutic prospects. *Current Neuropharmacology*.

